# Device and Patient-Related Complications of Renal Denervation for Uncontrolled Hypertension: A Post-Marketing Analysis of the FDA MAUDE Database

**DOI:** 10.51894/001c.168160

**Published:** 2026-08-28

**Authors:** Sai Tharun Reddy Gopannagari, Kesava Manikanta Achuta, Divya Ganisetti, Shravya Pingili, Towfiqul Chowdhury, Chadi Saad

**Affiliations:** 1 Department of Internal Medicine Garden City Hospital, Michigan, USA; 2 Department of Internal Medicine Atal Bihari Vajpayee Institute of Medical Sciences & Dr Ram Manohar Lohia Hospital, New Delhi, India; 3 Department of Internal Medicine University of Central Florida/ HCA Florida Ocala Hospital, Florida, USA; 4 Department of Nephrology Garden City Hospital, Michigan, USA

**Keywords:** Renal denervation, hypertension, MAUDE database, post-marketing surveillance, Device-related complications

## Abstract

**Introduction:**

Renal denervation (RDN) has re-emerged as an adjunctive therapy for uncontrolled hypertension following recent U.S. Food and Drug Administration (FDA) approvals of ultrasound- and radiofrequency-based systems. Although randomized trials report a favorable safety profile, post-marketing data may reveal additional safety signals in real-world settings.

**Methods:**

We performed a retrospective descriptive observational study of reports in the FDA Manufacturer and User Facility Device Experience (MAUDE) database from inception through December 22, 2025, which represented the final date of data extraction. Reports were identified using product code “QYI” and brand names “Paradise” and “Symplicity Spyral.” Eligible reports describing RDN for hypertension management were manually reviewed and categorized as vascular, device-related, hemodynamic/arrhythmic, neurologic, or other events.

**Results:**

A total of 33 reports were identified, including 19/33 (57.6%) involving the Paradise system and 14/33 (42.4%) involving the Symplicity Spyral system. Vascular complications were the most frequently reported events, accounting for 15/33 reports (45.5%), including renal artery dissection (5/33, 15.2%), thrombosis (3/33, 9.1%), vasospasm (3/33, 9.1%), pseudoaneurysm (2/33, 6.1%), renal hematoma (1/33, 3.0%), and acute limb ischemia (1/33, 3.0%). Device-related events accounted for 10/33 reports (30.3%), most commonly expired catheter use (4/33, 12.1%) and catheter or component malfunction (6/33, 18.2%). Hemodynamic/arrhythmic events occurred in 4/33 reports (12.1%), neurologic events in 2/33 (6.1%), and other events accounted for 2/33 reports (6.1%).

**Conclusions:**

In this descriptive MAUDE analysis, RDN was associated with uncommon but clinically relevant vascular and device-related adverse events in real-world practice. These findings support continued post-marketing surveillance as use of RDN expands. Interpretation is limited by the passive nature of MAUDE reporting, including underreporting, incomplete data, and the inability to determine incidence or causality.

## INTRODUCTION

Resistant hypertension is defined as failure to lower systolic and diastolic blood pressure despite lifestyle interventions and 3 or more antihypertensive drugs, including a diuretic, or if the patient is taking 4 or more antihypertensive drugs, irrespective of their blood pressure. It is strongly associated with increased all-cause mortality and major cardiovascular adverse events, including myocardial infarction, congestive heart failure, or stroke.^1^ This has prompted the search for more effective ways to manage drug-resistant hypertension, with renal denervation (RDN) demonstrating clinical benefit in selected patients. RDN is a minimally invasive, catheter-based intervention designed to attenuate sympathetic overactivity by disrupting renal sympathetic nerves located along the renal arteries. The procedure is typically performed via femoral arterial access under fluoroscopic guidance, with delivery of either radiofrequency (RF) energy or ultrasound energy to the renal arterial wall, where the sympathetic nerve fibers reside. By reducing sympathetic tone, RDN aims to achieve sustained reductions in blood pressure in patients with resistant or difficult-to-control hypertension.^2^ The 2023 European Society of Hypertension guidelines for the management of arterial hypertension recommended considering RDN as a treatment option in patients with uncontrolled blood pressure despite the use of antihypertensive drug combination therapy, or if a patient developed serious side effects from the drug therapy, or in a patient with resistant hypertension.^3^

In November 2023, two RDN systems, the Medtronic Symplicity Spyral System and the Recor Medical Paradise System, were approved by the U.S. Food and Drug Administration (FDA) for adjunctive treatment in patients with hypertension in whom lifestyle and antihypertensive medications do not adequately control blood pressure.^4^ Randomized clinical trials related to RDN, including the SPYRAL HTN program and the RADIANCE trials, have demonstrated sustained blood pressure reduction with a favorable safety profile and a low incidence of major adverse events.^5,6^ Clinical trials are typically conducted in highly controlled settings with carefully selected patients and experienced operators. In contrast, post-marketing surveillance databases such as the Manufacturer and User Facility Device Experience (MAUDE) offer a broader view of device performance and complications encountered in routine clinical practice. Therefore, we conducted a retrospective descriptive analysis of reports in the FDA MAUDE database to characterize device-related and patient-related adverse events associated with RDN for uncontrolled hypertension in real-world clinical practice.

## METHODS

### Study design and data source

We conducted a retrospective descriptive observational study of adverse event reports submitted to the US FDA Manufacturer and User Facility Device Experience (MAUDE) database, a passive surveillance system that captures suspected device-related adverse events and malfunctions. The MAUDE database was the sole data source for this study. The database was queried from inception through December 22, 2025, which represented the final date of data extraction.

### Search strategy and case selection

Reports were identified using the MAUDE search interface with product code “QYI” (Ablation Catheter, Renal Denervation) and device brand names including “Paradise” and “Symplicity Spyral.” We included reports describing RDN procedures performed for uncontrolled or resistant hypertension.^7^ Reports not related to RDN for hypertension or lacking sufficient information to confirm procedural relevance were excluded. Each retrieved report was manually reviewed for eligibility. When follow-up narratives were available within the same MAUDE event, they were reviewed together as part of a single report.

### Data extraction and event classification

For each eligible report, we extracted device manufacturer, event type, and narrative details describing patient-related and device-related complications. The primary outcome was the type of adverse event associated with RDN procedures. Events were grouped into prespecified categories: (1) vascular complications, (2) device-related complications, (3) hemodynamic/arrhythmic events, (4) neurologic events, and (5) other events. If more than one adverse event occurred in a single report, the most clinically significant event was designated as the primary complication category, and secondary events were described narratively. Reports were also reviewed for apparent duplicates; no definite duplicate reports were identified.

### Ethical considerations

This study used a publicly available, de-identified database and did not involve direct patient contact or access to protected health information; therefore, institutional review board approval was not required.

### Statistical analysis and reporting

Given the descriptive nature of MAUDE, only summary statistics were used. Categorical variables are presented as counts and percentages. No comparative statistical testing was performed. Consistent with FDA guidance, MAUDE data were used for safety signal identification only and were not used to estimate incidence, compare event rates between devices, or establish causality. STROBE principles were used to guide manuscript preparation, as applicable to this retrospective descriptive observational study.

## RESULTS

**Figure 1.**
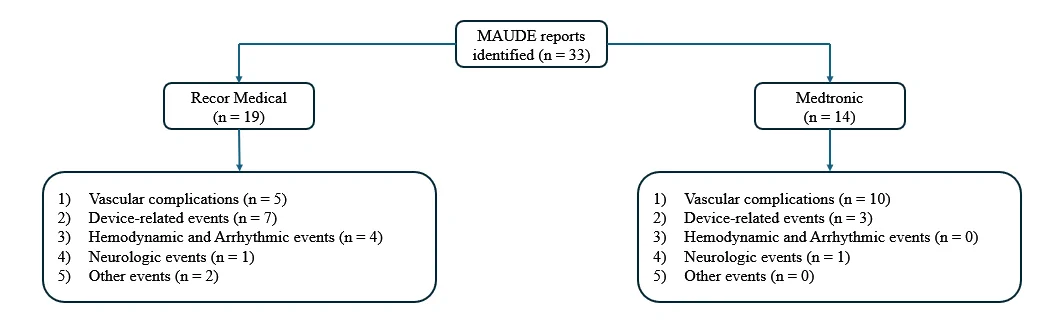
Classification of MAUDE-reported adverse events after RDN procedures by device manufacturer Flowchart showing the classification of 33 MAUDE-reported adverse events associated with RDN procedures, stratified by device manufacturer. Each report was assigned to one primary complication category based on the most clinically significant event described in the report. Secondary events, when present, are described separately in Table 1. Abbreviations: MAUDE, Manufacturer and User Facility Device Experience; RDN, renal denervation.

A total of 33 MAUDE reports related to RDN were identified. No apparent duplicate reports were identified on manual review. Of these, 19/33 (57.6%) involved the Paradise ultrasound system (Recor Medical), and 14/33 (42.4%) involved the Symplicity Spyral radiofrequency (RF) system (Medtronic) (Figure 1 and Table 1). The specific adverse events identified are described below:

### Vascular complications

Vascular complications were the most frequently reported adverse events, comprising 15/33 (45.5%) reports. Renal artery dissection occurred in five cases—two with the Paradise system (one associated with concurrent vasospasm) and three with the Symplicity Spyral system, all managed with balloon angioplasty and/or stent placement. Renal artery thrombosis was reported in three cases: one with the Paradise system, associated with tortuous anatomy and incomplete treatment, and two with the Symplicity Spyral system, which required thrombolysis and/or thrombectomy. Renal artery vasospasm was observed in three cases across both systems and was typically managed with intra-arterial nitroglycerin, with or without balloon dilation. Two cases of pseudoaneurysm were reported with the Symplicity Spyral system, including one access-site pseudoaneurysm complicated by hemorrhagic shock. Other vascular events included a renal parenchymal hematoma and one episode of acute limb ischemia, both associated with the Symplicity Spyral system.

### Device-related complications

Device-related events accounted for 10/33 (30.3%) reports. Expired catheter use was reported in four cases with the Paradise system; all procedures were completed without immediate clinical consequences. Catheter or guidewire malfunctions were noted in three cases, including kinking, resistance during wire advancement, or catheter deformation. One case involved device component detachment or migration with the Symplicity Spyral system, necessitating snare retrieval of a distal electrode. Additionally, entrapment of a non-manufacturer accessory was reported during a Symplicity Spyral procedure, and one case involved logistical or storage-related concerns with the Paradise system.

### Hemodynamic, neurologic, and other events

Hemodynamic/arrhythmic events occurred in 4/33 (12.1%) reports, all associated with the Paradise system, including bradycardia (two cases), transient hypoxia (one case), and transient asystole requiring brief resuscitation (one case). Neurologic events were uncommon, occurring in 2/33 (6.1%) reports; one of the patients developed a thunderclap headache and transient visual symptoms days after the procedure, without radiographic evidence of stroke or hemorrhage, and the other had syncope. Other events accounted for 2/33 (6.1%) reports and included patient dissatisfaction due to persistent hypertension despite the procedure, and one report with insufficient information.

**Table 1.** Device- and patient-related adverse events reported to the FDA MAUDE database after RDN procedures

**Manufacturer**	**Primary Complication Category**	**Specific adverse events**	**n**
Recor	Vascular	Renal artery dissection	2
Recor	Vascular	Renal artery thrombosis	1
Recor	Vascular	Renal artery vasospasm	2
Recor	Device-related	Expired catheter use	4
Recor	Device-related	Catheter or guidewire malfunction	1
Recor	Device-related	Missing device component	1
Recor	Device-related	Storage-related issue	1
Recor	Hemodynamic/arrhythmic	Bradycardia	2
Recor	Hemodynamic/arrhythmic	Transient asystole/cardiac arrest	1
Recor	Hemodynamic/arrhythmic	Transient hypoxia	1
Recor	Neurologic	Headache with transient visual symptoms	1
Recor	Other	Patient dissatisfaction	1
Recor	Other	Insufficient information	1
Medtronic	Vascular	Renal artery dissection	3
Medtronic	Vascular	Renal artery thrombosis	2
Medtronic	Vascular	Renal artery vasospasm	1
Medtronic	Vascular	Renal or access site pseudoaneurysm	2
Medtronic	Vascular	Renal parenchymal injury	1
Medtronic	Vascular	Acute limb ischemia	1
Medtronic	Device-related	Catheter or guidewire malfunction	1
Medtronic	Device-related	Device component detachment	1
Medtronic	Device-related	Device entrapment	1
Medtronic	Neurologic	Syncope	1

Summary of device- and patient-related adverse events reported to the FDA MAUDE database after RDN procedures. Reports reflect real-world post-marketing surveillance and may include multiple complications per patient; however, each report was assigned to a primary complication category based on the most clinically significant event. Data are descriptive and are not intended to estimate complication rates, compare safety between devices, or establish causality.

Abbreviations: FDA, U.S. Food and Drug Administration; MAUDE, Manufacturer and User Facility Device Experience; RDN, renal denervation.

## DISCUSSION

In this descriptive post-marketing analysis of the FDA MAUDE database, vascular complications were the most commonly reported adverse events associated with RDN, followed by device-related events. Although the total number of reports was small, these findings highlight the types of clinically relevant safety signals that may arise in routine practice outside the tightly controlled conditions of randomized trials.

Because MAUDE is designed for post-marketing surveillance, it captures suspected adverse events and device-related concerns but does not include data on uneventful procedures. In addition, although RDN has entered clinical practice following FDA approval, reliable publicly available post-approval utilization data remain limited. We did not identify reliable public data reporting the total number of U.S. RDN procedures performed, or the number of Paradise and Symplicity Spyral systems used, through the final MAUDE data extraction date of December 22, 2025. The absence of a procedural denominator limits quantitative risk assessment and reinforces that the events identified in this study should be interpreted as post-marketing safety signals in the setting of expanding but incompletely quantified clinical adoption, rather than as estimates of event frequency, device-specific safety comparisons, or causality.

The most prominent safety signal in this analysis was the occurrence of vascular complications, including renal artery dissection, thrombosis, vasospasm, pseudoaneurysm formation, and access-site–related events. Although these complications were uncommon and often managed with endovascular treatment or supportive care, they remain clinically important when they occur. Device-related events such as expired catheter use, catheter malfunction, and device entrapment further suggest that real-world safety considerations extend beyond patient anatomy alone and also include procedural and equipment-related factors.

A meta-analysis published in 2024 demonstrated a clinically significant reduction in both systolic and diastolic blood pressure with RDN compared with sham control.^8^ Pooled analyses from the RADIANCE clinical trial program and data from the Global Paradise System (GPS) Registry, presented at the 2025 Transcatheter Cardiovascular Therapeutics (TCT) conference, showed sustained reductions in office systolic blood pressure of approximately 15.7 mmHg for up to 24 months following ultrasound-based RDN using the Paradise system. Five major adverse events were reported in 3 of 293 patients (1.0%) (RADIANCE II, RADIANCE-HTN SOLO, and RADIANCE-HTN TRIO trials) in the treatment arm. One patient developed a pseudoaneurysm that was treated with intravenous thrombin injection, while another experienced a postprocedural vasovagal episode that resolved with fluid administration and atropine.^6,9–11^ Similarly, the SPYRAL HTN-ON MED trial demonstrated that RF-based RDN with the Symplicity Spyral system produced clinically meaningful and sustained blood pressure reductions through 36 months of follow-up, independent of concomitant antihypertensive therapy. Among 38 patients in the treatment arm, one patient had a hypertensive crisis and stroke at 427 days after the procedure, with no instances of renal artery perforation or dissection requiring intervention.^5^

These findings should be interpreted in the context of the different purposes and structures of randomized clinical trials and post-marketing surveillance. Randomized trials provide protocolized safety and efficacy data in selected study populations, whereas MAUDE reports describe suspected adverse events and device-related concerns submitted during real-world use. Therefore, the MAUDE findings should be viewed as complementary safety signals rather than as evidence of higher complication rates or direct comparative safety differences between clinical trials and routine practice.

Our findings are consistent with previously published literature evaluating adverse events associated with RDN.^12,13^ A Cochrane Library review analyzing 15 randomized controlled trials reported complications similar to those identified in our analysis, including vascular events such as renal artery dissection, vasospasm, and femoral artery pseudoaneurysm, as well as arrhythmic events like bradycardia and neurologic symptoms, including syncope.^14^ The overlap between previously reported complications and those identified in MAUDE supports the clinical and procedural plausibility of these adverse events, even when their overall frequency remains low.

Taken together, these findings complement randomized trial data by describing the types of vascular and device-related events reported during post-marketing use of contemporary RDN systems. Within the MAUDE reports analyzed, vascular complications represented the most frequently reported event category, while severe neurologic injury or fatal outcomes were not prominent safety signals. Continued post-marketing surveillance, careful patient selection, attention to vascular anatomy, adherence to device protocols, and procedural caution remain important as RDN use expands in routine clinical practice.

## LIMITATIONS

This study has several limitations inherent to the MAUDE database. First, MAUDE is a passive surveillance system and is subject to underreporting, stimulated reporting, and variable report quality. Second, many reports contain limited clinical detail, restricting assessment of baseline patient characteristics, operator experience, procedural technique, timing of events, management, and outcomes. Third, because MAUDE does not include the total number of devices used or procedures performed, incidence rates cannot be calculated, and safety events cannot be compared quantitatively between device platforms. Fourth, causality cannot be established, as reported events may reflect patient factors, procedural complexity, or incomplete narrative information rather than confirmed device failure alone. Fifth, duplicate or overlapping reports may occur in MAUDE, although no definite duplicates were identified on manual review in the present study. Finally, event classification required interpretation of narrative descriptions and may therefore be subject to investigator judgment.

## CONCLUSIONS

This descriptive analysis of MAUDE reports identified vascular and device-related events as the predominant post-marketing safety signals reported after RDN procedures. Because MAUDE lacks procedural denominators and cannot establish causality, these findings should not be interpreted as incidence estimates or comparative safety rates between device platforms. Instead, they provide a descriptive overview of reported event patterns that may help contextualize real-world procedural and device-related concerns as RDN is incorporated into routine hypertension care.

### Corresponding Author

Sai Tharun Reddy Gopannagari, MD

Department of Internal Medicine

Garden City Hospital, Michigan, USA

Email: saitharunreddygopannagari6@gmail.com

ORCID ID: 0009-0003-4015-1903

### Conflicts of interest

The authors declare no conflicts of interest related to this work.
